# Reducing Ion Transport Friction by Mitigated Diluent‐Solvent Interaction for Sodium–Sulfur Battery

**DOI:** 10.1002/advs.77440

**Published:** 2026-08-27

**Authors:** Sihang Xia, Jia Xu, Songling Wu, Shanshan Cao, Zhanju Wang, Xiangyang Gu, Jiang Liang, Kangning Zhao, Weina Xu, Chao Yang

**Affiliations:** ^1^ School of Environmental and Chemical Engineering Shanghai University Shanghai People's Republic of China; ^2^ School of Physical Sciences Great Bay University Dongguan People's Republic of China; ^3^ Zhongtian Technology Group Co., Ltd. Nantong People's Republic of China; ^4^ School of Materials Science and Engineering Dongguan University of Technology Guangdong People's Republic of China

**Keywords:** ether‐based electrolytes, localized high concentration, room‐temperature, sodium–sulfur batteries, solid‐electrolyte interphase

## Abstract

Sodium sulfur batteries have emerged as a promising candidate for large‐scale energy storage, while their practical implementation is severely hindered by sodium dendrite growth, unstable solid‐electrolyte interphase (SEI) films, and the polysulfide shuttle effect. Herein, we design a novel local high‐concentration electrolyte (LHCE‐DEE/TTEE) to balance the ionic conductivity and interfacial stability. This is achieved by replacing the high solvating DME with the moderate solvating diethyl ether (DEE) to mitigate the diluent‐solvent interaction and reduce the local dynamic friction for Na^+^ transport. Additionally, the replacement of DME with DEE weakens the ion‐dipole interaction to form stable anion‐enriched solvation clusters. Furthermore, the long ethyl chains in DEE provide sufficient steric hindrance to confine sulfur species within the cathode and suppress the polysulfide shuttle effect. Benefiting from these synergistic advantages, the Na||Na symmetric cell in LHCE‐DEE/TTEE sustains stable cycling for 2500 h. When coupled with a sulfurized polyacrylonitrile cathode, the designed electrolyte demonstrates robust stability at practical conditions of high sulfur loadings of 8.7 mg cm^−2^ and lean electrolyte of 4.6 µL mg^−1^ and this is further validated in pouch cell configurations. This work offers new understanding towards LHCE electrolyte design strategy to balance the ionic conductivity and interfacial stability.

## Graphical abstract

A local high‐concentration electrolyte to balance the ionic conductivity and interfacial stability is developed through replacing the high solvating DME with the moderate solvating diethyl ether to mitigate the diluent‐solvent interaction and reduce the local dynamic friction for Na^+^ transport, while maintaining the anion‐rich solvation clusters and the polysulfide suppression effect.

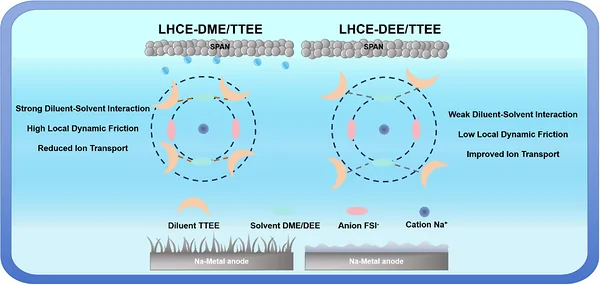

## 1 Introduction

Against the backdrop of the global carbon neutrality strategy and the rapid development of large‐scale energy storage technologies, sodium‐ion batteries have emerged as a core candidate system for next‐generation stationary energy storage and low‐speed power devices [1, 2, 3, 4, 5, 6, 7], owing to their abundant sodium reserves, low cost, and high compatibility with lithium‐ion battery manufacturing processes [8, 9, 10, 11]. Among them, the sodium metal anode, with its outstanding advantages of ultra‐high theoretical specific capacity (1166 mAh g^−1^) and extremely low redox potential (−2.71 V vs. standard hydrogen electrode) [12, 13, 14], is expected to completely break the capacity bottleneck of traditional intercalation anodes and construct high‐energy‐density sodium metal batteries. However, the sodium metal anode faces critical challenges during charging and discharging, including irreversible sodium dendrite growth, continuous electrolyte decomposition side reactions, and inhomogeneous and unstable solid electrolyte interphase (SEI) films. These issues not only drastically reduce the Coulombic efficiency and cycle life of the battery but also easily trigger safety hazards such as internal short circuits, severely restricting the practical application of sodium metal batteries [15, 16, 17], meanwhile, for high‐energy systems such as sodium–sulfur batteries, the shuttle effect of polysulfide intermediates further exacerbates anode interface degradation and cathode active material loss, creating a dual dilemma of dendrite growth and shuttle effect, which has become a key technical barrier to the commercialization of sodium metal batteries [18, 19, 20, 21, 22, 23].

As an ion transport medium connecting the cathode and anode, the solvation structure, component ratio, and interface interaction mechanism of the electrolyte directly determine the sodium deposition/stripping behavior, SEI film composition, and stability [24, 25, 26]. It is a core breakthrough for regulating interface reactions [27], inhibiting dendrite growth [28], and alleviating polysulfide shuttling [29]. Traditional dilute ether‐based electrolytes (e.g., dimethoxyethane (DME)‐based systems) exhibit good ionic conductivity, but solvent molecules have excessively strong coordination with Na^+^ [30], tending to form a dense solvent‐dominated solvation shell. This prevents anions from participating in the first solvation layer, resulting in SEI films rich in organic components with insufficient mechanical strength that cannot resist the penetration of sodium dendrites [31]. Meanwhile, strong polar solvents have high solubility for polysulfides and cannot effectively suppress the shuttle effect, further accelerating battery performance decay [32]. High‐concentration electrolytes (HCEs) construct an anion‐dominated solvation structure by increasing salt content, which can induce the formation of dense inorganic SEI films and achieve partial dendrite inhibition [33]. However, defects such as high viscosity, low ion migration rate, and high raw material cost limit their large‐scale application. On this basis, localized high‐concentration electrolytes (LHCEs) retain the advantageous solvation structure of the high‐concentration core region while reducing system viscosity and cost by introducing inert fluorinated ether diluents [34]. The model system of LHCE for Na‐S battery involves sodium bis(fluorosulfonyl)imide (NaFSI) as the sodium salt, hexafluoroisopropyl 2,2,2‐trifluoroethyl ether (TTEE) as the inert diluent, and long‐chain 1,2‐diethoxyethane (DEE) as the functional solvent. TTEE is regarded as inert to participate in the Na^+^ solvation structure, while its interaction could be ignored. However, despite the increase in interfacial stability, the LHCEs suffer from the low ionic conductivity and low ion transference number [35], compared to HCEs, which in turn compensates the rate capability. Therefore, it is crucial to balance the ionic conductivity and interfacial stability to achieve high‐performance Na–S battery.

Here, we show that by reducing the diluent‐solvent interaction through replacing the high‐solvating DME with moderate solvating DEE, the local dynamic friction would be greatly reduced to improve the Na^+^ ionic conductivity, while maintaining high anion‐dominated solvation to promote interfacial stability of the sodium anode. This forms an unencumbered fluorous matrix that acts as a low‐friction lubricating fluid, leaving the internal core of the ion channels highly mobile. Furthermore, the replacement of DME with DEE weakens the ion‐dipole interaction between solvent molecules and Na^+^, forcing more FSI^−^ anions into the first solvation shell of Na^+^ to form stable anion‐enriched solvation clusters and ensured the NaF‐rich interface in sodium metal anode. Meanwhile, its electron‐withdrawing effect reduces the solubility of polysulfide intermediates, blocks their shuttle channels between the cathode and anode, alleviates performance degradation caused by the shuttle effect, and achieves dual regulation of anode interface protection and cathode system stability. In this way, the LHCE‐DEE/TTEE shows higher ionic conductivity lean electrolyte compared to LHCE‐DME/TTEE (1.61 vs. 0.61 mS/cm). Benefiting from these features above, the Na||Na symmetric cell in LHCE‐DEE/TTEE sustains stable cycling for 2500 h. When coupled with a sulfurized polyacrylonitrile (SPAN) cathode, the designed electrolyte demonstrates robust stability at practical conditions of high SPAN loadings of 8.7 mg cm^−2^ and lean electrolyte of 4.6 µL mg^−1^ and this is further validated in pouch cell configurations. Our work presents a new design principle for high‐performance LHCE electrolyte to balance the ionic conductivity and interfacial stability.

## 2 Results and Discussions

### 2.1 Molecular Design and Solvation Structure Regulation of the Electrolyte

The localized high‐concentration electrolyte was formulated with NaFSI as the sodium salt, TTEE as the inert diluent, and long‐chain DEE with steric hindrance as the functional solvent. For comparison, strongly coordinating DME and TTEE, as well as pure DEE, were used as solvents separately. To investigate the effect of DEE on solvation coordination, electrostatic potential simulations were performed on the three molecules [36]. This electrolyte possesses greater steric hindrance, which elevates the electron density around the solvent molecules, thereby weakening the solvent‐polysulfide interactions and reducing polysulfide dissolution compared with DEE (Figure 1). As shown in Figure 2a, in the electrostatic potential map of the DME molecule, the ether oxygen atom region shows a prominent negative potential with a large range, indicating that ether oxygen atoms are the core electron‐donating sites. This strong coordination causes DME to preferentially encapsulate Na^+^ in the solvation system, forming a solvent‐dominated solvation shell and inhibiting FSI^−^ anions from entering the first solvation layer. In contrast, compared with DME, the long‐chain ethyl side chains of DEE bring obvious steric hindrance, shrinking the blue electronegative region around the ether oxygen atoms [37]. This is attributed to the steric hindrance effect “shielding” the electron cloud of the ether oxygen atoms, directly weakening the electron‐donating ability and coordination activity of oxygen atoms and reducing the coordination strength between DEE and Na^+^. This weakened coordination frees up solvation space for FSI^−^ anions, promoting anions to participate in the first solvation shell of Na^+^ and constructing a stable anion‐enriched solvation structure. TTEE, as a diluent, exhibits electrochemical inertness. This electronic inertness prevents TTEE from forming stable coordination bonds with Na^+^, making it an ideal inert diluent. Meanwhile, the electron‐withdrawing property of F in TTEE reduces polysulfide solubility, blocking polysulfide shuttle channels at the molecular level. Furthermore, frontier molecular orbital analysis revealed significant differences in electronic stability among different solvation structures (Figure 2b). The LUMO energy level of the Na^+^FSI^−^‐TTEE cluster is the lowest (−0.99 eV), significantly lower than that of Na^+^FSI^−^‐DME (−0.64 eV) and Na^+^FSI^−^‐DEE (−0.68 eV). A lower LUMO energy level implies a higher tendency for reductive decomposition [38], indicating that the TTEE‐based solvation structure preferentially decomposes on the sodium metal surface, facilitating the formation of an inorganic‐rich SEI film and laying the foundation for dendrite inhibition.

**FIGURE 1:**
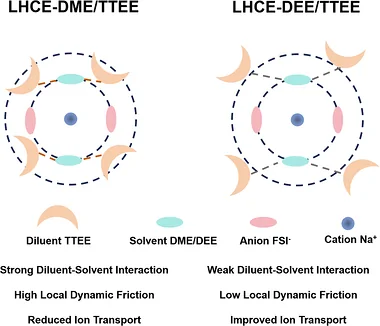
Schematic diagrams of Na−S batteries in different electrolytes.

**FIGURE 2:**
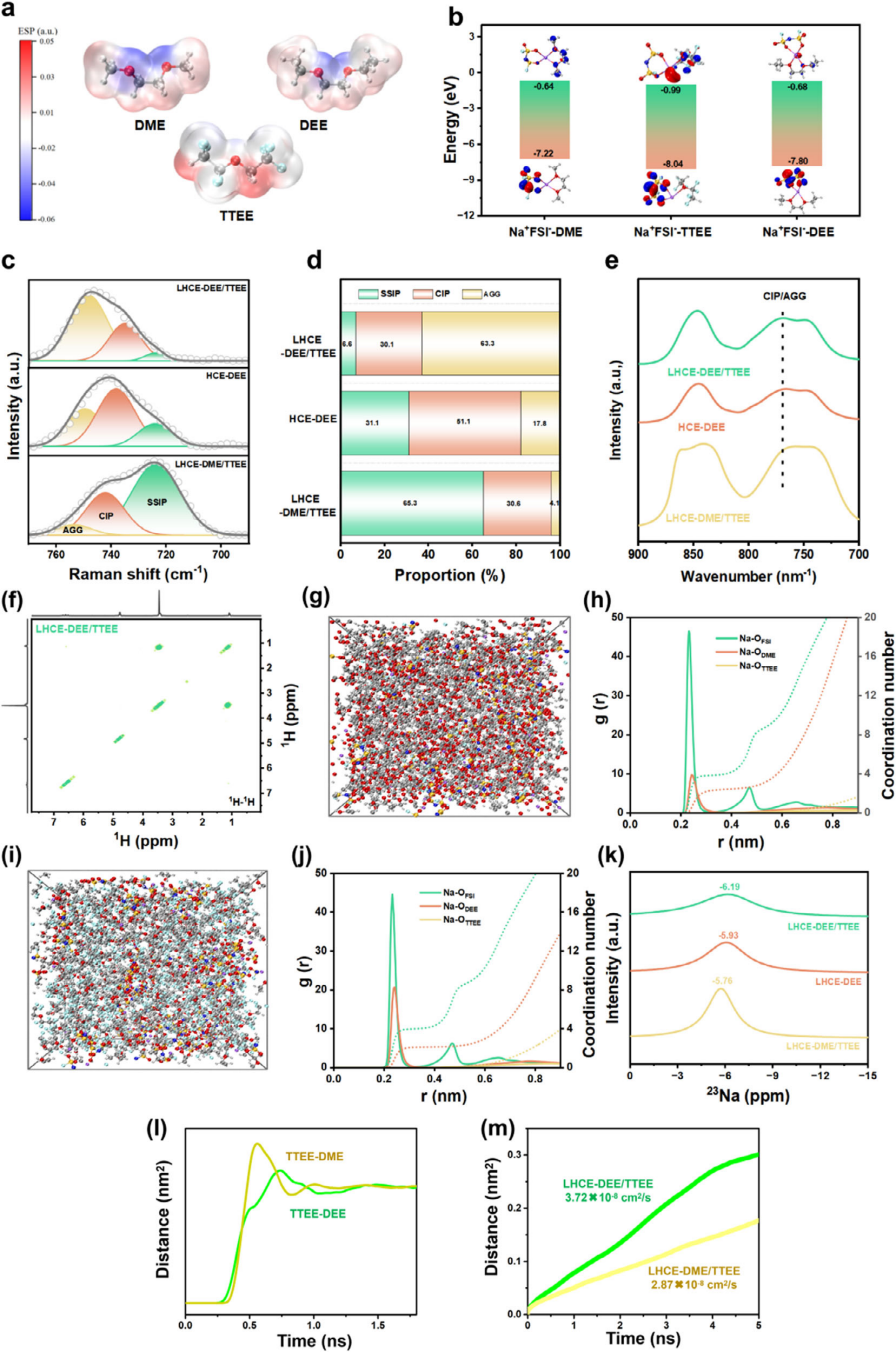
(a) Electrostatic potential (ESP) of different solvents and their corresponding binding energies with Na‐ion. (b) HOMO and LUMO energy levels of different solvents with Na ion. (c) Deconvoluted Raman spectra of different electrolytes. (d) Contents of SSIP, CIP, and AGG in different electrolytes. (e) FT‐IR spectra of different electrolytes. (f)1H–1H COSYNMR spectra of LHCE‐DEE/TTEE. (g) MD simulation snapshots of LHCE‐DME/TTEE. (h) RDFs of Na^+^ centered components in LHCE‐DME/TTEE. (i) MD simulation snapshots of LHCE‐DEE/TTEE. (j) RDFs of Na^+^ centered components in LHCE‐DEE/TTEE. (k) ^23^Na NMR spectra of various electrolytes. (l) RDFs of TTEE centered components in LHCE‐DME/TTEE and LHCE‐DME/TTEE. (m) Self‐diffusion rate of in LHCE‐DME/TTEE and LHCE‐DME/TTEE.

Raman spectroscopy was further used to study the solvation structures of the three electrolyte systems (Figure 2c). Specifically, the S–N–S stretching signal of free FSI^−^ anions in the sodium salt can be deconvoluted into three characteristic peaks: SSIP (723 cm^−1^, corresponding to free FSI^−^), CIP (742 cm^−1^, one FSI^−^ coordinated with one Na^+^), and AGG (754 cm^−1^, one FSI^−^ coordinated with two or more Na^+^). As shown in the figure, LHCE‐DEE/TTEE has the least SSIP, originating from the weak coordination of DEE with sodium ions, which promotes the formation of an anion‐rich solvation shell around Na^+^ [39]. In contrast, in LHCE‐DME/TTEE, the strong interaction between DME and sodium ions reduces the coordination number between sodium and FSI^−^, leading to the generation of more‐free FSI^−^ [40]. The relative contents in Figure 2d further confirm this. In addition, Fourier‐transform infrared spectroscopy was used to analyze the solvation structures of different localized high‐concentration electrolytes. Figure 2e shows that the CIP/AGG peak at 769 cm^−1^ of the LHCE‐DEE/TTEE electrolyte becomes sharper and more pronounced compared with HCE‐DEE, indicating an increased proportion of contact ion pairs and aggregate structures [41]. These results collectively demonstrate that Na^+^ and FSI^−^ share a solvation shell rather than existing in independent solvation environments. Such an anion‐rich environment favors the formation of an inorganic‐phase‐rich SEI film on the sodium metal anode. 2D ^1^H–^1^H correlation spectroscopy was further used to characterize solvent–solvent interactions (Figure 2f, Figure S1a,b). The horizontal and vertical axes represent the ^1^H NMR signals of the electrolyte, and cross‐peaks originate from ^1^H–^1^H coupling, reflecting the proximity and signal interference of hydrogen dipoles, thus characterizing both intramolecular and intermolecular coupling signals. Intramolecular interactions along the diagonal are stronger due to the shorter distance between intramolecular hydrogen dipoles. Intermolecular ^1^H–^1^H coupling stems from solvent–solvent interactions (δH^+^‐δO^−^) [42], shortening the distance between solvent molecules and overlapping hydrogen electron clouds. This enhancement is attributed to the ion‐dipole interaction between Na^+^ and the solvent. Compared with LHCE‐DME/TTEE, LHCE‐DEE/TTEE exhibits stronger solvent–solvent interactions, which facilitates Na^+^ desolvation.

Molecular dynamics simulations were further performed to study the sodium ion solvation structures in LHCE‐DME/TTEE and LHCE‐DEE/TTEE [43]. As shown in Figure 2g–j and Figure S2a,b, in the LHCE‐DME/TTEE electrolyte system, the coordination numbers of sodium ions with FSI^−^, and DME are 3.84 and 2.27, respectively. In LHCE‐DEE/TTEE, the coordination numbers are 3.98 and 2.07, respectively. The increased coordination number between sodium ions and FSI^−^ and the lower coordination number with DEE further confirm that DEE promotes the coordination between sodium ions and anions. In the LHCE‐DEE/TTEE system (Figure 2k), the strongest high‐field shift is −6.19 ppm, indicating a very dense electron cloud around Na^+^, a typical characteristic of anion‐dominated CIP and AGG [44, 45]. The solvation structure of TTEE is further examined in Figure 2l, and the interaction of LHCE‐DEE/TTEE is much weaker than that of LHCE‐DME/TTEE. Furthermore, the self‐diffusion rate of LHCE‐DEE/TTEE is much higher than that of LHCE‐DME/TTEE in Figure 2m, suggesting that the local dynamic friction is greatly reduced due to weaker interaction of Na^+^ solvation structure with the diluent. We calculated the Na^+^ clusters with the diluent along with the MD simulations, and it is found that in LHCE‐DEE/TTEE electrolyte, the TTEE shows half coordination with that in LHCE‐DME/TTEE electrolyte (Figure S3). Along with the improved ionic conductivity, it suggests that the lower pair interaction energy distributions reduce the friction and improve the ionic conductivity.

### 2.2 Electrochemical Performance of LHCE‐DEE/TTEE

Coulombic efficiency (CE) is a key indicator for evaluating the electrochemical stability of sodium metal anodes during repeated cycling [46]. Half‐cells assembled with LHCE‐DEE/TTEE, LHCE‐DME/TTEE, and HCE‐DEE electrodes were tested at a current density of 0.5 mA cm^−2^ to verify the effects of dendrite inhibition and cycle stability improvement. The modified Aurbach method was used to accurately measure the average Coulombic efficiency of Na||Cu batteries during Na^+^ stripping/deposition (Figure 3a). The average CE of LHCE‐DEE/TTEE reaches 97.1%, much higher than that of HCE‐DEE (94.6%) and LHCE‐DME/TTEE (80.4%). This is attributed to its excellent Na^+^ transport performance and stable SEI film, which effectively prevent dead sodium formation and continuous solvent decomposition [47]. Furthermore, the cycle rate stability of Na||Cu half‐cells assembled with LHCE‐DEE/TTEE electrolyte was tested at different current densities (1–2 mA cm^−2^) (Figure 3b). It can be seen that even under different currents, LHCE‐DEE/TTEE‐based batteries maintain stable cycling and high Coulombic efficiency, owing to the anion‐rich layer that stabilizes SEI formation and promotes uniform sodium ion deposition. LHCE‐DEE/TTEE not only improves the Coulombic efficiency of sodium ions but also enhances sodium ion migration efficiency. Figure 3c shows that the sodium ion transference number of LHCE‐DME/TTEE‐based batteries is 0.52, because the strong interaction between oxygen atoms in DME and Na hinders Na^+^ desolvation to a certain extent. The transference number of LHCE‐DEE/TTEE electrolyte increases to 0.57 (Figure S4). In contrast, LHCE‐DEE/TTEE‐based batteries exhibit the highest sodium ion transference number of 0.66 (Figure 3d,e).

**FIGURE 3:**
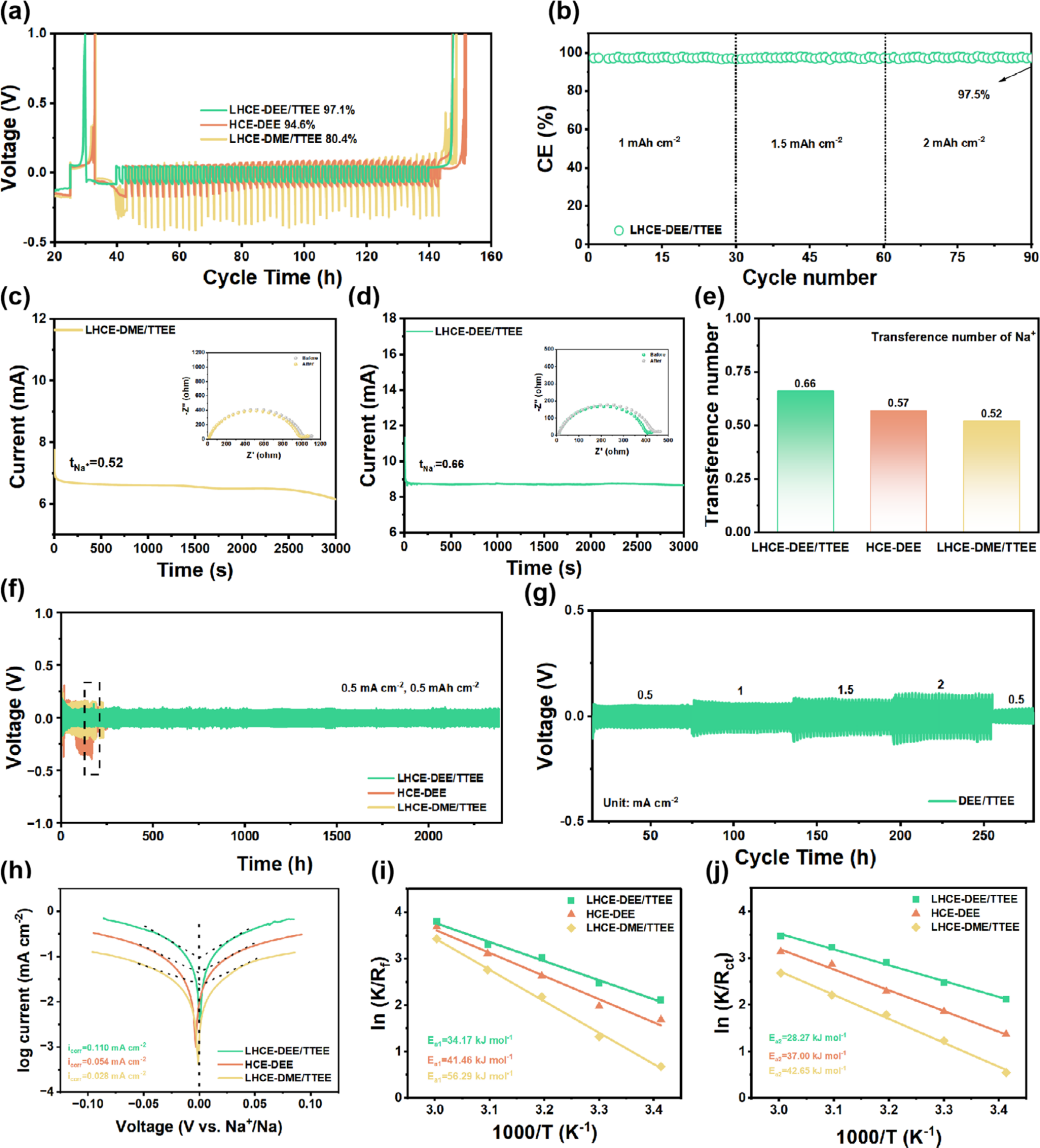
(a) Average CE measurements of Na−Cu cells cycled with various electrolytes at 0.5 mA/0.5 mAh cm^−2^. (b) Voltage profiles and CE of Na–Cu cells cycled at 1, 1.5 and 2 mAh cm^−2^. Nyquist plots of the cells with (c) LHCE‐DME/TTEE, (d) LHCE‐DEE/TTEE, (e) The ion transference number in different electrolytes. (f) Cycling performance of Na||Na symmetric cells with different electrolytes at 0.5 mA cm^−2^/0.5 mAh cm^−2^. (g) Rate performance of Na||Na symmetric cell with different electrolytes. (h) Tafel plot of Na||Na symmetric cells with various electrolytes. Activation energies for (i) R_f_ and (j) R_ct_ derived from the Nyquist plots of Na||Na symmetric cells cycled with different electrolytes.

Na||Na symmetric cells were assembled to further evaluate the effect of LHCE‐DEE/TTEE‐based electrolyte on promoting uniform sodium deposition and stripping [48]. Galvanostatic charge/discharge tests were conducted on symmetric cells assembled with different electrolytes. Figure 3f shows the time‐voltage curves of symmetric cells at a current density of 0.5 mA cm^−2^. The voltage polarization of LHCE‐DME/TTEE‐based battery continuously intensifies and short‐circuits after approximately 200 h. In contrast, LHCE‐DEE/TTEE‐based batteries cycle stably for 2500 h, and the voltage fluctuation is significantly smaller than that of LHCE‐DME/TTEE and HCE‐DEE‐based batteries. Typically, sodium deposition is faster at higher current densities, making dendrite formation more likely. Therefore, the current was further increased to verify the stability of LHCE‐DEE/TTEE‐based symmetric cells under high currents. As shown in Figure S5a,b, when the current density rises to 1 and 2 mA cm^−2^, the cells cycle stably for 1000 and 800 h, respectively. In addition, the rate performance of symmetric cells was tested at different current densities from 0.5 to 2 mA cm^−2^. As shown in Figure 3g, LHCE‐DEE/TTEE‐based batteries exhibit small voltage fluctuations at any current density, confirming that the anion‐dominated solvation structure formed by LHCE‐DEE/TTEE effectively inhibits battery polarization and endows symmetric cells with good rate performance. Furthermore, the positive effects of LHCE‐DEE/TTEE‐based electrolyte on Na diffusion kinetics and desolvation were verified. The Tafel curve in Figure 3h shows that the exchange current density of LHCE‐DEE/TTEE‐based batteries (0.11 mA cm^−2^) is higher than that of HCE‐DEE batteries (0.054 mA cm^−2^) and LHCE‐DME/TTEE‐based batteries (0.028 mA cm^−2^), indicating that LHCE‐DEE/TTEE enhances Na^+^ diffusion and reduces Na^+^ migration barriers. EIS analysis at different temperatures further confirms the positive role of LHCE‐DEE/TTEE. As shown in Figure S6, the impedance of LHCE‐DEE/TTEE‐based batteries is lower than that of the other two electrolytes at all temperatures. As shown in Figure 3i,j and Tables S1 and S3, the Arrhenius equation was used to calculate the energy barriers for Na^+^ crossing the SEI (corresponding to R_f_) and desolvation (corresponding to R_ct_) layer in LHCE‐DEE/TTEE‐based electrolyte [49], which are 34.17 and 28.27 kJ mol^−1^, respectively, significantly lower than those of the other two electrolytes. This reduction in energy barriers further implies that LHCE‐DEE/TTEE forms a more ion‐conductive and dense SEI layer, promoting the desolvation of Na^+^ and rapid ion transfer kinetics.

Full cells were assembled by matching with sulfurized polyacrylonitrile cathodes to characterize the feasibility of LHCE‐DEE/TTEE‐based electrolyte in practical battery operation. As shown in Figure 4a and Figure S7, with a cathode loading of 3.5 mg/cm^2^ and an electrolyte/SPAN (E/SPAN) ratio of 11.4 µL mg^−1^, LHCE‐DEE/TTEE electrolyte‐based batteries retain a capacity of 861.82 mAh g^−1^ after 500 cycles at 0.2C (capacity retention rate of 99.6%). In contrast, full cells assembled with HCE‐DEE and LHCE‐DME/TTEE electrolytes degrade to 680.71 and 457.61 mAh g^−1^ after 500 cycles, respectively. The current was further increased to 0.5 C and 1 C, and LHCE‐DEE/TTEE electrolyte‐based batteries still maintain excellent performance (Figure 4b,c, Figure S8). To meet high‐energy‐density requirements, the battery performance was further tested under practical conditions involving high sulfur loading (SPAN loading = 8.7 mg cm^−2^) and low electrolyte usage (E/SPAN = 4.6 µL mg^−1^) (Figure 4d). It can be seen that LHCE‐DEE/TTEE electrolyte‐based batteries still retain a capacity of 845.66 mAh g^−1^ after 1000 cycles. In addition, the rate performance of the batteries was tested at different current densities (0.1C–1C) (Figure 4e). The results show that at current densities of 0.5C, 1C, 2C, 5C, and 0.2C, the capacities of LHCE‐DEE/TTEE‐based batteries are 999.30, 967.92, 885.78, 852.73, and 822.47 mAh g^−1^, respectively; those of HCE‐DEE‐based batteries are 973.70, 812.66, 747.77, 704.32, and 658.18 mAh g^−1^; and those of LHCE‐DME/TTEE are 612.84, 484.68, 477.49, 432.14, and 413.53 mAh g^−1^. The performance comparison in this work is shown in Figure 4f and Table S4 [42, 50, 51, 52, 53, 54, 55]. Capacity–voltage curves further confirm the high capacity of LHCE‐DEE/TTEE (Figure 4g,h, Figure S9). To further verify the practicality of the LHCE‐DEE/TTEE‐based electrolyte system, pouch cells were assembled and tested for long‐cycle performance (Figure 4i), retaining an excellent capacity of 592.01 mAh g^−1^ after 80 cycles. These results indicate that by introducing DEE with steric hindrance, LHCE‐DEE/TTEE‐based batteries exhibit excellent full‐cell performance under various conditions.

**FIGURE 4:**
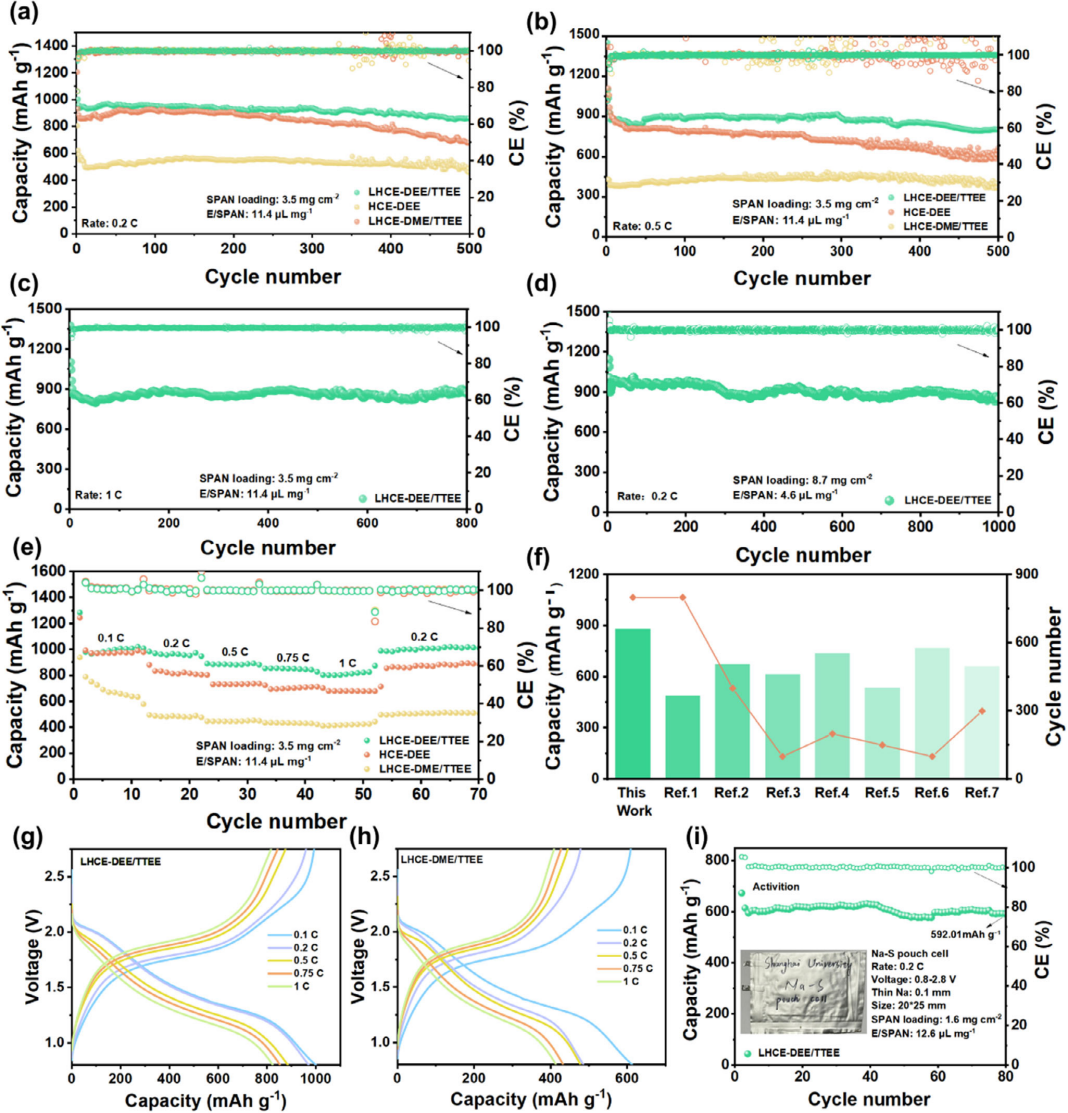
Cycle performances of Na‐S cells with different electrolyte at (a) 0.2C, (b) 0.5C, (c) 1C. (d) High sulfur loading Na‐S cells with LHCE‐DEE/TTEE electrolyte at 0.2C rate. (e) Rate performances of Na‐S cells with different electrolyte. (f) Comparison of our work with recently reported Na‐S cells. Rate capabilities of Na‐S cells with discharge−charge curves with (g) LHCE‐DEE/TTEE, (h) LHCE‐DME/TTEE. (i) Digital photos and cycle performance of Na‐S pouch cell with LHCE‐DEE/TTEE electrolyte at 0.2C.

### 2.3 Na^+^ Deposition and Polysulfide Inhibition Mechanism

Optical images were used to observe the sodium metal morphology of LHCE‐DEE/TTEE, HCE‐DEE, and LHCE‐DME/TTEE‐based batteries after 50 cycles. As shown in Figure 5a–c, due to the anion‐dominated solvation shell in the LHCE‐DEE/TTEE‐based electrolyte, uniform SEI components are formed on the sodium surface, so the LHCE‐DEE/TTEE‐based electrode surface exhibits metallic luster and a smooth surface. SEM images further clearly confirm this: the LHCE‐DEE/TTEE electrode surface shows a dense structure. Compared with HCE‐DEE and LHCE‐DME/TTEE‐based electrolytes, LHCE‐DEE/TTEE effectively induces uniform sodium ion deposition, thereby stabilizing the sodium metal anode (Figure 5d–f). XPS was used to further characterize the SEI components of the sodium metal anode.

**FIGURE 5:**
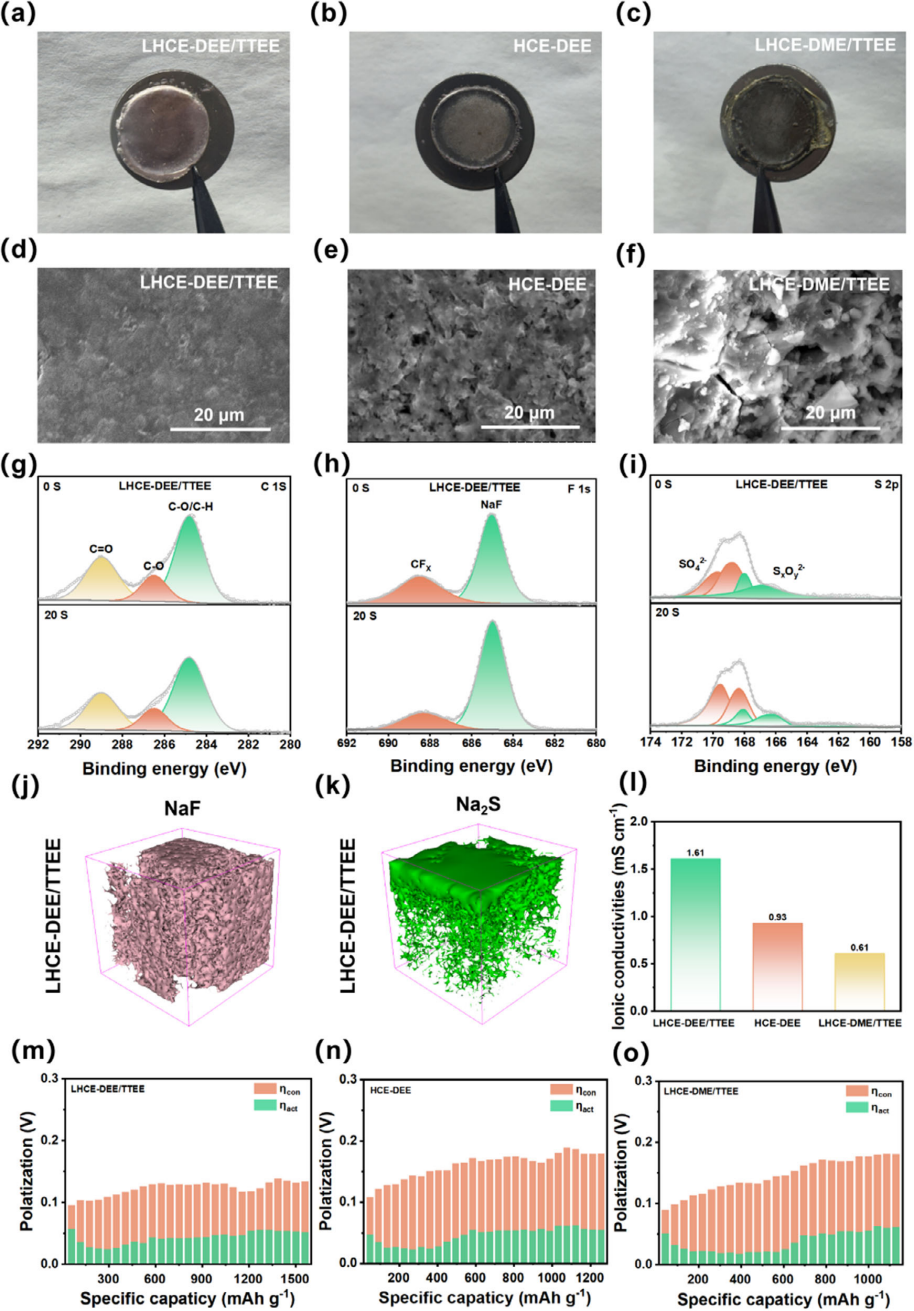
Digital photos of the cycled Na‐metal anodes from Na||Na symmetric cells with (a) LHCE‐DEE/TTEE, (b) HCE‐DEE, and (c) LHCE‐DME/TTEE. SEM images of cycled sodium‐metal anodes from Na‐S cells with (d) LHCE‐DEE/TTEE, (e) HCE‐DEE, and (f) LHCE‐DME/TTEE. (g–i) C 1s, F 1s and S 2p XPS spectra of the cycled sodium metal anode under LHCE‐DEE/TTEE. (j,k) The TOF‐SIMS 3D reconstruction of the NaF and Na_2_S components of the cycled sodium metal anode in LHCE‐DEE/TTEE. (l) Ionic conductivity of different electrolytes. (m–o) Changes of η_con_ and η_act_ in the cell with different electrolytes during the initial discharge process.

XPS was used to further characterize the SEI components of the sodium metal anode [56]. The C 1s spectrum shows that the outer layer of the SEI film formed by LHCE‐DEE/TTEE is mainly composed of C─C/C─H (284.8 eV), C─O (286.6 eV), and C═O (288.9 eV) (Figure 5g), originating from the decomposition of ether solvents. The intensity of the C─C peak gradually decreases during sputtering, indicating the varying degrees of solvent reduction. The F 1s spectrum shows that the NaF content in the LHCE‐DEE/TTEE system is the highest among the three systems (Figure 5h), confirming the formation of an inorganic‐rich SEI film on the sodium metal anode. It has been reported that NaF improves ionic conductivity and SEI stability [57]. Meanwhile, in the S 2p spectrum (Figure 5i), the strongest signals are oxidized sulfur species (SO_4_2^−^ and S_x_Oᵧ^2−^) and reduced sulfide species. The XPS spectra of HCE‐DEE and LHCE‐DME/TTEE are shown in Figures S10 and S11, respectively. These results indicate that the SEI film is mainly produced by the reductive decomposition of anions and the fluorinated TTEE diluent. Furthermore, time‐of‐flight secondary ion mass spectrometry (TOF‐SIMS) depth profiling (Figure 5j,k) was performed on the cycled anode of the LHCE‐DEE/TTEE system to spatially reconstruct the spatial composition and structural uniformity of the SEI layer [58]. 3D reconstruction shows a clear distribution of inorganic components. As shown in Figure 5j,k, the signals of inorganic components, namely NaF and NaS_2_, are dense and uniformly distributed in the sputtered material. The highest NaF content in LHCE‐DEE/TTEE is attributed to DEE promoting the binding of anions to sodium ions via weak coordination, so anions are preferentially reduced. This ensures high ionic conductivity of the SEI layer and endows it with certain mechanical flexibility to resist sodium dendrite growth.

SEM and EDS measurements were adopted to observe the surface condition, morphology, and elemental distribution of cycled SPAN cathodes under different electrolyte environments, as illustrated in Figures S12–S14. The LHCE‐DEE/TTEE sample shows the smoothest electrode surface with merely 8.0% sodium content, while the HCE‐DEE electrode turns slightly rough with 11.4% sodium. Noticeable cracks emerge on the LHCE‐DME/TTEE cathode accompanied by a high sodium proportion of 16.6%. Surface sodium enrichment originates from electrolyte oxidation by‐products and back‐deposited polysulfides caused by the shuttle effect. LHCE‐DEE/TTEE is capable of constructing a stable cathode interface to restrain solvent decomposition and polysulfide deposition, reduce interfacial side reactions, and stabilize electrode structure. However, DME with strong solvation capacity fails to immobilize soluble polysulfides and worsens the shuttle effect. Coupled with the weak antioxidant property of DME, substantial sodium‐rich byproducts accumulate heterogeneously, leading to mismatched interfacial stress and structural damage of SPAN electrodes.

Ionic conductivity tests were performed to evaluate the ion transport capability of different electrolyte systems. The results show that the LHCE‐DEE/TTEE electrolyte delivers a high ionic conductivity of 1.61 mS/cm, which is significantly higher than those of the HCE‐DEE (0.93 mS/cm) and LHCE‐DME/TTEE (0.61 mS/cm) electrolytes (Figure 5l and Figure S15). The superior ionic conductivity of LHCE‐DEE/TTEE benefits from its optimized solvation structure and reduced ion migration resistance. Such excellent ion‐transport properties facilitate rapid sodium ion migration within the electrolyte and electrode interface, effectively accelerating the electrochemical reaction kinetics of sodium–sulfur batteries.

To explore the effect of LHCE‐DEE/TTEE on the sulfur cathode, sodium–sulfur full cells were prepared with sulfurized polyacrylonitrile as the cathode, and their electrochemical performance and reaction kinetics were tested via cyclic voltammetry [59]. As shown in Figure S16a–d, when using LHCE‐DEE/TTEE electrolyte, two reduction peaks appear near 1.8 and 1.2 V in the battery, corresponding to the gradual reduction of sulfur in the sulfurized polyacrylonitrile framework to short‐chain sodium polysulfide intermediates (Na_2_S_x_, x ≤ 4) and the further sodiation of short‐chain sodium polysulfides to sodium sulfide, respectively. During oxidation, the oxidation peak near 1.9 V is attributed to the desodiation reaction of sodium sulfide and its re‐incorporation into the polyacrylonitrile framework. Notably, there is no additional oxidation peak near 2.3 V, indicating that almost no traditional solid‐liquid phase transition occurs in the sulfurized polyacrylonitrile cathode. This proves that the long‐chain DEE molecules in LHCE‐DEE/TTEE carry ethyl alkyl side chains that generate significant steric hindrance, thereby alleviating the polysulfide shuttle effect.

In addition, cyclic voltammetry tests at different scan rates (0.1–0.3 mV·s^−1^) were conducted to evaluate the battery reaction kinetics. The results show that at the same scan rate, compared with HCE‐DEE and LHCE‐DME/TTEE, the LHCE‐DEE/TTEE system has a higher response current and lower voltage polarization; as the scan rate increases, the current response of the LHCE‐DEE/TTEE cyclic voltammetry curve increases more significantly. According to the Randles–Sevcik equation, fitting the relationship between peak current and the square root of scan rate shows that the slope of the LHCE‐DEE/TTEE fitting curve is larger [60], indicating faster sulfur reduction kinetics in batteries using this electrolyte. The linear sweep voltammetry (LSV) curves presented in Figure S17 demonstrate the outstanding charge‐transfer kinetics and ultra‐broad electrochemical stability window of the LHCE‐DEE/TTEE electrolyte. The oxidation stability potential of this electrolyte reaches up to 4.57 V (vs Na/Na^+^). The improvement in electrochemical stability originates from the synergistic interaction between TTEE and DEE solvents, which endows the LHCE‐DEE/TTEE electrolyte with weak solvation capability. The electrolyte operates stably within the practical working voltage range of sodium–sulfur batteries (0.8–2.8 V), effectively avoiding internal short‐circuit failure of the cell. Compared with the HCE‐DEE and LHCE‐DME/TTEE electrolyte systems, LHCE‐DEE/TTEE delivers superior electrochemical stability over a broader voltage window.

Galvanostatic intermittent titration technique (GITT) was employed to quantitatively investigate the sodium storage kinetics of the SPAN cathode in different electrolyte systems, with the original test profiles provided in Figure S18. Based on the voltage response characteristics of each GITT pulse, the total electrode overpotential can be decomposed into three independent components: ohmic polarization(η_ohm_), concentration polarization (labeled as η_con_), and activation polarization (labeled as η_act_) [61, 62]. Specifically, ohmic polarization originates from the intrinsic ohmic resistance of the electrolyte, electrode materials, and current collectors, and manifests as an instantaneous voltage jump at the moment of current on/off. Concentration polarization arises from the mass transport limitation of active species between the bulk electrolyte and the electrode reaction interface, while activation polarization is dominated by the charge transfer energy barrier of the redox reaction at the electrode/electrolyte interface. Since the ohmic contribution remains relatively small and steady under the present conditions, the subsequent analysis is centered on η_con_ and η_act_, which represent the dominant kinetic limitations. Figure 5m–o, Figures S19 and S20 present the polarization decomposition results, illustrating the composition and evolution of concentration polarization and activation polarization during the initial discharge process [63]. By comparison, the LHCE‐DEE/TTEE electrolyte endows the SPAN cathode with the lowest overall polarization and the slowest growth rate of concentration polarization, indicating its superior interfacial charge transfer efficiency and ion mass transport capability. This favorable kinetic feature effectively alleviates polarization accumulation under high sulfur loading, thus ensuring stable cycling performance of the electrode.

To investigate the polysulfide shuttle effect originating from sodium polysulfide dissolution, the solubility of sodium polysulfide in different electrolytes was further studied. First, polysulfide solubility tests were performed to clarify the interaction between electrolytes and polysulfides: 0.02 m Na_2_S_6_ was added to pure mixed solvents without NaFSI. The results show (Figure S21) that compared with HCE‐DEE and LHCE‐DME/TTEE, the color of the Na_2_S_6_ solution in LHCE‐DEE/TTEE electrolyte is significantly lighter, indicating that these electrolytes effectively limit Na_2_S_6_ dissolution and reduce the polysulfide shuttle effect. The distribution of S species on the surface of the cycled sodium metal anode was further used to confirm the effect of LHCE‐DEE/TTEE on the polysulfide shuttle effect. As shown in Figures S22–S24, LHCE‐DME/TTEE has a high sulfur content of 14.6 wt.% and strong signals in the elemental mapping, indicating severe polysulfide corrosion. In contrast, the steric effect induced by DEE in LHCE‐DEE/TTEE effectively immobilizes sulfur and inhibits the polysulfide shuttle effect, achieving a sulfur content as low as 6.1 wt.%.

## 3 Conclusions

In summary, this study successfully designed and constructed a novel local high‐concentration electrolyte (LHCE‐DEE/TTEE) through mitigated diluent‐solvent interaction. By precisely regulating the solvation structure‐diluent interaction at the molecular level, it simultaneously addresses the dual challenges of ionic conductivity and interface stability, including sodium dendrite growth and the polysulfide shuttle effect in sodium metal batteries. In the LHCE‐DEE/TTEE electrolyte, DEE weakens the coordination between solvent molecules and Na^+^ and promotes the incorporation of anions into the primary solvation shell, enabling tailored regulation of the solvation structure while suppressing dendrite growth and polysulfide shuttling in a synchronous manner. This electrolyte facilitates the formation of a compact, inorganic‐rich SEI film, which significantly improves the uniformity of sodium deposition, Coulombic efficiency, cycling stability, and ion transport kinetics. Under high sulfur loading (8.7 mg cm^−2^) and low electrolyte dosage (E/SPAN = 4.6 µL mg^−1^), the LHCE‐DEE/TTEE‐based cell maintains a capacity of 845.66 mA h g^−1^ after 1000 cycles, accompanied by enhanced rate capability. This work provides new understanding of diluent and solvent interaction for the LHCE electrolyte design of dendrite‐free, highly stable sodium metal batteries and their practical application in energy storage.

## Author Contributions

**Sihang Xia**: data curation, investigation, validation, writing – original draft, formal analysis, conceptualization. **Jia Xu**: methodology, data curation, validation, investigation, writing – original draft. **Songling Wu**: methodology, formal analysis. **Shanshan Cao**: investigation, formal analysis. **Zhanju Wang**: investigation. Xiangyang Gu: investigation. **Jiang Liang**: validation. **Kangning Zhao**: conceptualization, methodology, formal analysis, writing – review and editing, writing – original draft, funding acquisition, investigation. **Weina Xu**: conceptualization, investigation, writing – review and editing, validation, formal analysis. **Chao Yang**: conceptualization, investigation, writing – original draft, writing – review and editing, project administration, formal analysis, supervision.

## Conflicts of Interest

The authors declare no conflicts of interest.

## Supporting information




**Supporting File**: advs77440‐sup‐0001‐SuppMat.docx.

## Data Availability

The data that support the findings of this study are available from the corresponding author upon reasonable request.
