# Supplementary material for: Reducing Ion Transport Friction by Mitigated Diluent‐Solvent Interaction for Sodium–Sulfur Battery
Source: Adv Sci (Weinh). 2026 Aug 27;13(63):e77440. doi: 10.1002/advs.77440 (PMC13521030; doi:10.1002/advs.77440)
Supplement: Supplementary file 1 [file ADVS-13-e77440-s001.docx]

**Supporting Information**

**Reducing Ion Transport Friction by Mitigated Diluent-Solvent Interaction for Sodium Sulfur Battery**

*Sihang Xia*^#^*, Jia Xu*^#^*, Songling Wu*, *Shanshan Cao, Zhanju Wang, Xiangyang Gu, Jiang Liang, Kangning Zhao*, Weina Xu*, and Chao Yang**

S. Xia, J. Xu, S. Wu, C. Yang

School of Environmental and Chemical Engineering, Shanghai University, Shanghai 200444, China

Email: chaoyang@shu.edu.cn

J. Liang, K. Zhao

School of Physical Sciences, Great Bay University, Dongguan, China

Email: vicyel@gbu.edu.cn

S. Cao, Z. Wang, X. Gu

Zhongtian Technology Group Co.,Ltd., Nantong Economic and Technical Development Area, Nantong 226400, China

W. Xu

School of Materials Science and Engineering, Dongguan University of Technology,

Guangdong 523808, P. R. China

Email: xuwn@dgut.edu.cn

^#^These authors contributed equally to this work.

**Experimental Section**

**Materials：**

1,2-dimethoxyethane (DME, Dodochem),ethylene glycol diethyl ether(DEE, Adamas）,and 1,1,2,2-tetrafluoroethyl-2,2,2-trifluoroethyl ether (TTEE, Aladdin) were dried with molecular sieves for at least 3 days. Sodium bis(fluorosulfonyl)imide (NaFSI, Dodochem) was dissolved separately in mixed solvents DME/TTEE (v:v = 5:5), DEE, and DEE/TTEE (v:v = 5:5) to obtain 2M LHCE-DME/TTEE, 2M HCE-DEE, and 2M LHCE-DEE/TTEE electrolytes. All the electrolytes were prepared inside an argon-filled glove box.

**Synthesis of sulfurized polyacrylonitrile (SPAN)**

Commercial-grade sulfur and polyacrylonitrile (both provided by Aladdin) were initially ball-milled in a 4:1 mass ratio in an isopropanol environment for 12 hours to obtain a uniformly dispersed powder mixture. After the ball-milling process was completed, the composite was transferred to a drying oven and maintained at 40 °C for 12 hours to remove residual solvent. The dried powder was then loaded into a tube furnace and heat-treated under argon protection at a heating rate of 2 °C per minute, with a final temperature of 350 °C maintained for 6 hours. After naturally cooling to room temperature, the obtained sulfur-polyacrylonitrile (SPAN) product was collected and used to fabricate SPAN working electrodes. The sulfur content in the as-prepared SPAN is determined to be ~45 wt% by elemental analysis.

**Assembly of the cells**

CR2032 coin-type cells were fabricated in an Ar-filled glove box, where sodium metal served as the anode; the oxygen and water contents inside the glove box were maintained below 0.1 ppm. Ceramic-coated Celgard 2500 was employed as the separator. The electrolytes were prepared following the above-mentioned procedures, and the electrolyte/SPAN (E/SPAN) ratio in the coin-type cells was ~11.4 μL mg^-1^. For a high sulfur loading of 8.7 mg cm^-2^, the E/SPAN ratio was 4.6 μL mg^-1^.

The ceramic-coated Celgard 2500 separator was selected for its superior wettability, reducing electrolyte volume to 40 μL per coin-type cell (E/SPAN ratio = 11.4 μL mg^-1^). This not only lowers electrolyte usage, but also enhances the energy density of the batteries.

In addition to coin cells, pouch cells were fabricated in this work for practical validation. Aluminum foils coated with SPAN at an areal loading of 1.6 mg cm^-2^ were cut into 20 mm × 25 mm pieces to serve as cathodes, while sodium metal foil with a thickness of 0.1 mm and dimensions of 20 mm × 25 mm was used as the anode. The electrolyte-to-SPAN mass ratio (E/SPAN) was fixed at 12.6 μL mg^-1^. Galvanostatic charge-discharge cycling tests of the pouch cells were carried out at a rate of 0.2 C within a voltage window of 0.8–2.8 V (vs Na^+^/Na). Notably, both the coin cells mentioned earlier and the newly prepared pouch cells were assembled in the same argon-filled glove box, where the concentrations of water and oxygen were strictly kept below 0.1 ppm to prevent detrimental side reactions triggered by moisture and oxygen.

**Electrochemical characterizations**

Cycling performance data were acquired using a Land cycler system, with tests carried out at room temperature within a voltage window of 0.8 to 2.8 V.^[1]^ Prior to formal testing, the cells underwent a precycling process at a 0.05C rate for one cycle to stabilize their electrochemical performance. The polarization behavior of Na||Na symmetric cells and the Coulombic efficiency of Na-Cu asymmetric cells were examined under different deposition/stripping capacity conditions. For all tests in this study, the specific capacity values were calculated based on the mass of sulfur in the cathode.

In this study, the Arrhenius model was employed to fit and calculate temperature-varying impedance parameters including interfacial resistance (R_f_) and charge transfer resistance (R_ct_) collected via electrochemical impedance spectroscopy (EIS), through which the apparent activation energies matching each key electrochemical reaction were quantified. Special focus was placed on analyzing sodium ion desolvation, sodium ion penetration through solid electrolyte interphase (SEI) films, as well as the ion migration across the boundary between SEI layers and electrode substrates. The mathematical form of the Arrhenius relationship is written as follows:

k = A exp (−E_a_/RT) (1)

where k corresponds to the reaction rate constant; A refers to the pre-exponential factor (alternatively named frequency factor), which correlates with the collision frequency among reactive species. Ea denotes activation energy, namely the minimum energy barrier required to trigger electrochemical reactions. R stands for the universal gas constant with a fixed value of 8.314 J/(mol·K), and T represents the absolute thermodynamic temperature measured in Kelvin (K).

The electrochemical stability windows of the as-prepared electrolytes were quantitatively evaluated through linear sweep voltammetry (LSV) tests conducted at a constant temperature of 25 °C.^[2]^ In these LSV measurements, a stainless steel (SS) blocking electrode served as the working electrode, whereas a sodium metal foil acted as both the counter electrode and the reference electrode simultaneously. The LSV scans were performed on a Bio-logic electrochemical workstation, with the potential sweeping from the open-circuit potential up to 5 V (versus Na^+^/Na) at a moderate scan rate of 5 mV s^-1^, ensuring the capture of complete oxidation behavior information. A capacitive baseline of 0 mA cm^-2^ was set in this work. The intersection between the rising segment of the oxidation current curve and the capacitive baseline was defined as the oxidation onset potential of electrolytes to obtain reliable electrochemical window parameters.

The ionic conductivities of the prepared electrolytes were evaluated through electrochemical impedance spectroscopy (EIS) tests, conducted over a frequency range from 100 kHz to 1 Hz. These measurements were performed using a VMP3 multichannel electrochemical workstation (produced by Bio-logic), with an alternating current amplitude set at 5 mV to ensure the accuracy and reliability of the test results. To conduct the conductivity measurements, dedicated test cells were assembled by submerging two stainless steel blocking electrodes into the electrolyte samples.The ionic conductivity of electrolyte samples was calculated via the formula listed below:

σ = l / RA (2)

In this formula, the variables are defined as follows: σ stands for ionic conductivity, l refers to the thickness of the tested membrane, A represents the effective contact area of stainless steel electrodes, and R denotes the bulk resistance of the electrolyte.

For galvanostatic intermittent titration technique (GITT) characterizations,^[3][4]^ the battery cells underwent an initial activation process: one complete charge-discharge cycle at a rate of 0.05C to stabilize the internal electrochemical state. Subsequent GITT measurements were performed by subjecting the pre-activated cells to a constant-current charge-discharge step at 0.1C for 15 minutes, followed by a 60-minute rest period at open-circuit voltage (OCV) in each cycle. To capture all subtle variations in voltage and capacity profiles that might occur during the titration process, the experimental data were sampled and recorded at 10-second intervals throughout the entire testing procedure.

Cyclic voltammetry (CV) measurements were performed at scan rates ranging from 0.1 to 0.3 mV s^-1^, covering a voltage range of 0.8 to 2.8 V.^[5]^ Electrochemical impedance spectroscopy (EIS) analyses were conducted on a Biologic electrochemical workstation, with the test frequency spanning from 100 kHz down to 1 Hz.

**Characterizations**

Fourier Transform Infrared (FTIR) spectroscopy was implemented in the attenuated total reflectance (ATR) mode, with measurements carried out on a Thermo Scientific-Nicolet iS5 FTIR spectrometer.^[6]^ The structural features of the as-prepared electrolytes were characterized via Raman spectroscopy using an Alpha 300 Witec Micro-Raman spectrometer. ¹H-¹H correlation spectroscopy (COSY, with 4 scans and 256 t₁ increments) was performed to further characterize the electrolytes.^[7]^

Morphological observations were performed using a field emission scanning electron microscope (FEI Quanta 650 SEM, operated at an accelerating voltage of 20 kV), coupled with an energy dispersive spectrometer (EDS) for elemental signal detection.^[8]^ X-ray photoelectron spectroscopy (XPS) analysis was conducted at room temperature on a Thermo Scientific K-Alpha spectrometer, utilizing monochromatic Al Kα radiation (1,486.6 eV) as the excitation source.^[9]^ To avoid air exposure, the cells were disassembled inside an argon-filled glove box, and the harvested samples were transferred into the XPS analysis chamber using a sealed argon-filled container. Additionally, The acquired XPS spectra were processed using CasaXPS software: following Shirley background subtraction, the spectral peaks were deconvoluted by fitting the experimental data with multiple Gaussian-Lorentzian functions.^[10]^ Time-of-flight secondary ion mass spectrometry (TOF-SIMS) tests with high ion detection sensitivity were carried out on a TOF-SIMS V instrument (ION-TOF GmbH),^[11]^ with a sputtering time set to 600 s. The raw TOF-SIMS data were subsequently processed and visualized using TOF-DR software.

**Theoretical Calculations**

Density functional theory (DFT) calculations were performed using the Gaussian16 software package. The ground-state molecular geometries were optimized at the B3LYP/6-311G+(d,p) theoretical level,^[12, 13]^ and the energy levels of the highest occupied molecular orbital (HOMO), lowest unoccupied molecular orbital (LUMO), as well as the molecular electrostatic potential surfaces, were evaluated at the same level.^[14, 15]^

The initial configurations of the target systems were constructed via the PACKMOL software package. Ab initio molecular dynamics (AIMD) simulations were executed using version 2023.1 of the CP2K program.^[16]^ All computational processes employed the PBE density functional theory incorporated with D3(BJ) dispersion correction to ensure calculation accuracy.^[17]^ To lower the demand for cut-off energy, the sodium element was computed using the all-electron pob-TZVP basis set,^[18]^ whereas the remaining elements were calculated with the DZVP-MOLOPT-SR-GTH basis set paired with the GTH-PBE pseudopotential.^[19]^

Prior to formal simulations, the prepared systems were first subjected to energy minimization through optimization calculations, with a plane-wave energy cut-off set at 400 Ry. The convergence thresholds for both the inner and outer self-consistent field (SCF) density matrices were fixed at 1.0×10⁻⁵. All simulations were carried out within an 18×18×18 Å^3^ periodic box, utilizing the Canonical sampling through velocity rescaling (CSVR) thermostat. Each system was simulated under an NVT ensemble at 333.15 K for a total duration of 10 ps, with an integration time step of 1 fs and a time constant of 200 fs.

**Figure S1.** 1H-1H COSY NMR spectra of (a) HCE-DEE, (b) LHCE-DME/TTEE.

**Figure S2.** (a) Coordination numbers of Na^+^ with O_FSI_ and O_DEE_ in HCE-DEE electrolytes. (b) Coordination numbers of Na^+^ with O_FSI_ and O_DME_ in LHCE-DME/TTEE electrolytes.

**Figure S3.** (a) Variation curve of DEE coordination number centered on TTEE. (b) Variation curve of DME coordination number centered on TTEE.

**Figure S4.** Nyquist plots of the cells with HCE-DEE.

**Figure S5.** Cycling performance of Na||Na symmetric cells with LHCE-DEE/TTEE at (a) 1 mA cm^−2^/1 mAh cm^−2^. (a) 2 mA cm^−2^/1 mAh cm^−2^.

**Figure S6.** Nyquist plots of the cells at different temperatures with (a) LHCE-DEE/TTEE. (b) HCE-DEE. (c) LHCE-DME/TTEE

**Figure S7.** Galvanostatic discharge-charge profiles of Na-S cells with (a) LHCE-DEE/TTEE, (b) HCE-DEE, and (c) LHCE-DME/TTEE at 0.2 C.

**Figure S8.** Galvanostatic discharge-charge profiles of Na-S cells with (a) LHCE-DEE/TTEE, (b) HCE-DEE, and (c) LHCE-DME/TTEE at 0.5 C.

**Figure S9.** Rate capabilities of Na−S cells with discharge−charge curves with LHCE-DME/TTEE.

**Figure S10.** (a) C 1s, (b) F 1s and (c) S 2p XPS spectra of the cycledsodium metal anode under HCE-DEE.

**Figure S11.** (a) C 1s, (b) F 1s and (c) S 2p XPS spectra of the cycled sodium metal anode under LHCE-DME-TTEE.

**Figure S12.** (a) SEM micrographs, (b) Na elemental mapping, (c) S elemental mapping, and (d) matched EDS spectra of Na anodes retrieved from Na–S cells cycled at 0.5 C in LHCE-DEE/TTEE electrolyte.

**Figure S13.** (a) SEM micrographs, (b) Na elemental mapping, (c) S elemental mapping, and (d) matched EDS spectra of Na anodes retrieved from Na–S cells cycled at 0.5 C in HCE-DEE electrolyte.

**Figure S14.** (a) SEM micrographs, (b) Na elemental mapping, (c) S elemental mapping, and (d) matched EDS spectra of Na anodes retrieved from Na–S cells cycled at 0.5 C in LHCE-DME/TTEE electrolyte.

**Figure S15**. Ionic conductivity of coin cells with various electrolytes was measured via EIS at room temperature.

**Figure S16.** CV curves of Na-S cells with (a) LHCE-DEE/TTEE, (b) HCE-DEE, and (c) LHCE-DME/TTEEBat different scan rates. (d) The linear relationship between peak currents and scan rates.

**Figure S17.** LSV measurements of different electrolytes.

**Figure S18.** GITT profiles for the cell with LHCE-DEE/TTEE electrolyte.

**Figure S19.** GITT profiles for the cell with (a) LHCE-DEE/TTEE, (b) HCE-DEE and (c) LHCE-DME/TTEE electrolyte.

**Figure S20.** (a) Activation polarization and (b) concentration polarization variations

versus the discharge process. (c) Activation polarization and (d) concentration polarization variations versus the charge process.

**Figure S21.** (a)Digital photographs of solubility tests for 0.02 M Na₂S₆ in (a) LHCE-DME/TTEE (b) HCE-DEE. (c) LHCE-DEE/TTEE.

**Figure S22.** (a-b) SEM images, (c) sulfur elemental mapping images, and (d) EDS spectra of Na-metal anodes disassembled from Na-S cells after 50 cycles at 0.5C rate with LHCE-DEE/TTEE electrolyte.

**Figure S23.** (a-b) SEM images, (c) sulfur elemental mapping images, and (d) EDS spectra of Na-metal anodes disassembled from Na-S cells after 50 cycles at 0.5C rate with HCE-DEE electrolyte.

**Figure S24.** (a-b) SEM images, (c) sulfur elemental mapping images, and (d) EDS spectra of Na-metal anodes disassembled from Na-S cells after 50 cycles at 0.5C rate with LHCE-DME/TTEE electrolyte.

**Table 1. Fitting values of the impedance spectra of a Na||Na symmetric cell with LHCE-DEE/TTEE electrolyte.**

| **T (K)** | **1000/T (K)** | **R_f_ (Ω)** | **R_ct_ (Ω)** |
| --- | --- | --- | --- |
| 333 | 3.0030 | 7.36 | 10.26 |
| 323 | 3.0960 | 11.91 | 12.78 |
| 313 | 3.1949 | 12.76 | 17.05 |
| 303 | 3.3003 | 25.38 | 25.38 |
| 293 | 3.4130 | 35.52 | 35.17 |

**Table 2. Fitting values of the impedance spectra of a Na||Na symmetric cell with HCE-DEE electrolyte.**

| **T (K)** | **1000/T (K)** | **R_f_ (Ω)** | **R_ct_ (Ω)** |
| --- | --- | --- | --- |
| 333 | 3.0030 | 8.32 | 14.41 |
| 323 | 3.0960 | 14.40 | 18.31 |
| 313 | 3.1949 | 22.60 | 31.70 |
| 303 | 3.3003 | 41.88 | 47.17 |
| 293 | 3.4130 | 54.07 | 74.44 |

**Table 3. Fitting values of the impedance spectra of a Na||Na symmetric cell with LHCE-DME/TTEE electrolyte.**

| **T (K)** | **1000/T (K)** | **R_f_ (Ω)** | **R_ct_ (Ω)** |
| --- | --- | --- | --- |
| 333 | 3.0030 | 10.78 | 22.83 |
| 323 | 3.0960 | 20.44 | 35.44 |
| 313 | 3.1949 | 35.39 | 52.26 |
| 303 | 3.3003 | 80.94 | 88.57 |
| 293 | 3.4130 | 148.44 | 169.04 |

**Table S4**. Comparative analyses of Na-S cells with LHCE-DEE/TTEE electrolyte and the previously reported Na-S electrolyte work

| **Electrolyte** | **Cathode** | **Cathode mass**  **loding**  **(mg cm^-2^)** | **Current**  **density** | **Reversible**  **Capcity**  **(mAh g^-1^)** | **Cycle number** | **Ref** |
| --- | --- | --- | --- | --- | --- | --- |
| LHCE-DEE/TTEE | SPAN | 3.5 | 1C | 881 | 800 | This  work |
| Mix-CPME | SPAN | 2.2 | 0.5C | 490 | 800 | [14] |
| 3.7MNaFSI in G2 | SPAN | 5.3 | 0.1C | 675 | 400 | [20] |
| SPAN+ZnS | SPAN+  ZnS | 4.0 | 0.14C | 618 | 100 | [21] |
| LHCE-Toluene | SPAN | / | 0.5C | 740 | 200 | [22] |
| 2.0M, W/DTD and Bi(OTf)_3_ | SPAN | 4.0 | 0.5C | 535 | 150 | [23] |
| LHCE-DMP/TFTFE | SPAN | 2.2 | 0.2C | 768 | 100 | [7] |
| LHCE-THP/ETFE | SPAN | 2.2 | 0.5C | 663 | 300 | [10] |
|  |  |  |  |  |  |  |

**References**

[1] J. Pampel, S. Dörfler, H. Althues, S. Kaskel. Designing room temperature sodium sulfur batteries with long cycle-life at pouch cell level[J]. Energy Storage Materials, 2019, 21: 41-49.
[2] J. Wu, Y. Tian, Y. Gao, Z. Gao, Y. Meng, Y. Wang, X. Wang, D. Zhou, F. Kang, B. Li, G. Wang. Rational electrolyte design toward cyclability remedy for room-temperature sodium–sulfur batteries[J]. Angewandte Chemie International Edition, 2022, 61(30): e202205416.
[3] K. Yang, P. Niu, Z. Feng, Y. Zhu, Z. Song, J. Meng. Rapid acquisition of battery impedance across multiple scenarios using drt analysis[J]. Journal of Energy Storage, 2024, 102:
[4] Y. Zhu, T. Gao, X. Fan, F. Han, C. Wang. Electrochemical techniques for intercalation electrode materials in rechargeable batteries[J]. Acc Chem Res, 2017, 50(4): 1022-1031.
[5] H. Park, H. Kim, J. M. Kim, H. Kang, S. Kansara, Z. Sun, M. Agostini, J. Rizell, A. Matic, S. Xiong, Y.-K. Sun, J.-Y. Hwang. Engineering of electrolyte solution in governing the stability of sodium metal anode for room temperature sodium–sulfur batteries[J]. ACS Energy Letters, 2026, 11(2): 1751-1760.
[6] W. Jan, A. D. Khan, F. J. Iftikhar, M. Z. Abbasi, G. Ali, E. Iwuoha, S. Shahzad, F. Ahmad, N. Ahmad. Electrolyte design for lithium-sulfur batteries: Progress and challenges[J]. Renewable and Sustainable Energy Reviews, 2025, 221:
[7] W. Yao, M. H. Pai, A. Manthiram. Inner-outer sheath synergistic shielding of polysulfides in asymmetric solvent-based electrolytes for stable sodium-sulfur batteries[J]. J Am Chem Soc, 2025, 147(14): 12061-12074.
[8] Y. Liu, S. Lu, S. Weng, J. Xu, H. Tu, Z. Wang, J. Xue, L. Liu, F. Zhang, G. Sun, Y. Gao, C. Qian, Z. Liu, H. Li, X. Wu. Interphase-regulated room-temperature sodium-sulfur batteries enabled by a nonflammable dual-functional electrolyte[J]. Advanced Energy Materials, 2025, 15(13): 2404890.
[9] A. Ghosh, S. Shukla, M. Monisha, A. Kumar, B. Lochab, S. Mitra. Sulfur copolymer: A new cathode structure for room-temperature sodium–sulfur batteries[J]. ACS Energy Letters, 2017, 2(10): 2478-2485.
[10] W. Yao, M. H. Pai, A. Manthiram. Deciphering the impact of polysulfide solvation structure on electrical double layer chemistry in sodium-sulfur batteries[J]. Angew Chem Int Ed Engl, 2025, 64(21): e202424547.
[11] B. Paudel, J. A. Dhas, Y. Zhou, M.-J. Choi, D. J. Senor, C.-H. Chang, Y. Du, Z. Zhu. Tof-sims in material research: A view from nanoscale hydrogen detection[J]. Materials Today, 2024, 75: 149-165.
[12] S. Grimme, S. Ehrlich, L. Goerigk. Effect of the damping function in dispersion corrected density functional theory[J]. Journal of Computational Chemistry, 2011, 32(7): 1456-1465.
[13] M. Kløve, S. Sommer, B. B. Iversen, B. Hammer, W. Dononelli. A machine-learning-based approach for solving atomic structures of nanomaterials combining pair distribution functions with density functional theory[J]. Advanced Materials, 2023, 35(13): 2208220.
[14] M. H. Pai, A. Manthiram. Fluorine‐free cosolvent chemistry empowering sodium‐sulfurized polyacrylonitrile batteries[J]. Advanced Energy Materials, 2025, 15(25):
[15] P. Peljo, H. H. Girault. Electrochemical potential window of battery electrolytes: The homo–lumo misconception[J]. Energy & Environmental Science, 2018, 11(9): 2306-2309.
[16] M. Iannuzzi, J. Wilhelm, F. Stein, A. Bussy, H. Elgabarty, D. Golze, A. S. Hehn, M. Graml, S. Marek, B. S. Gokmen, C. Schran, H. Forbert, R. Z. Khaliullin, A. Kozhevnikov, M. Taillefumier, R. Meli, V. V. Rybkin, M. Brehm, R. Schade, O. Schutt, J. V. Pototschnig, H. Mirhosseini, A. Knupfer, D. Marx, M. Krack, J. Hutter, T. D. Kuhne. The cp2k program package made simple[J]. J Phys Chem B, 2026, 130(4): 1237-1310.
[17] J. Proppe, S. Gugler, M. Reiher. Gaussian process-based refinement of dispersion corrections[J]. J Chem Theory Comput, 2019, 15(11): 6046-6060.
[18] A. Z. de Oliveira, C. T. Campos, F. E. Jorge, I. B. Ferreira, P. A. Fantin. All-electron triple zeta basis sets for the actinides[J]. Computational and Theoretical Chemistry, 2018, 1135: 28-33.
[19] Y. Wu, S. S. Liu, K. Y. Huang, Q. H. Yang, Y. Zheng, L. C. Li. Adsorption characteristics of sulfonamide antibiotic molecules on carbon nanotube and the effects of environment[J]. J Mol Model, 2023, 29(5): 150.
[20] X. Fan, Z. Zhong, X. Sun, Y. Wang, B. Chong, X. Kong, F. Li, L. Liu, J. Wang. Regulating solvating configuration to achieve long-cycle-life in sodium-span batteries[J]. Angew Chem Int Ed Engl, 2026, 65(15): e2019274.
[21] C. Sanjaykumar, Sungjemmenla, M. Chandra, C. B. Soni, S. K. Vineeth, S. Das, N. Cohen, H. Kumar, D. Mandler, V. Kumar. Tuning the local chemistry of span to realize the development of room-temperature sodium–sulfur pouch cells[J]. Journal of Materials Chemistry A, 2025, 13(2): 1420-1429.
[22] M. H. Pai, T. Lai, A. Manthiram. Sodium–sulfur cells with a sulfurized polyacrylonitrile cathode and a localized high concentration electrolyte with toluene as a nonfluorinated diluent[J]. Advanced Functional Materials, 2024, 34(44):
[23] Y. Ren, T. Lai, A. Manthiram. Reversible sodium–sulfur batteries enabled by a synergistic dual-additive design[J]. ACS Energy Letters, 2023, 8(6): 2746-2752.
